# From Therapy to Toxicity: A Rare Case of Acute Piperacillin/Tazobactam-Associated Drug-Induced Liver Injury

**DOI:** 10.7759/cureus.93434

**Published:** 2025-09-28

**Authors:** Priya Kumari Maheshwari, Alexis Maddox, Jun Yoo, Anil Harrison, Peter Fahim

**Affiliations:** 1 Department of Internal Medicine, University of Central Florida, Pensacola, USA; 2 Department of Internal Medicine, HCA Florida West Hospital, Pensacola, USA; 3 Department of Family Medicine, Alabama College of Osteopathic Medicine, Dothan, USA; 4 Department of Medicine, Midwestern University, Glendale, USA; 5 Department of Critical Care Medicine, University of Central Florida, Pensacola, USA; 6 Department of Critical Care Medicine, HCA Florida West Hospital, Pensacola, USA

**Keywords:** antibiotic-induced hepatotoxicity, beta-lactam antibiotic toxicity, drug-induced liver injury, hepatocellular injury, piperacillin/tazobactam

## Abstract

Drug-induced liver injury (DILI) is a diagnosis of exclusion, but one that requires high clinical suspicion because prompt withdrawal of the offending agent is imperative to improve prognosis. Piperacillin/tazobactam (TZP) is a rare cause of clinically apparent liver injury. This case is an example of DILI caused by TZP that was identified when a hospitalized patient’s liver enzymes greatly increased, and hepatic steatosis was subsequently seen on a computed tomography scan. The Roussel Uclaf Causality Assessment Method (RUCAM) score is a tool used to indicate the likelihood of DILI. In this case, the RUCAM score was 7, indicating that the causality assessment of the TZP as the cause of liver injury was probable. This case underscores the importance of maintaining a high index of suspicion for DILI, particularly in patients receiving extended courses of antibiotics, and highlights the need for routine liver function monitoring during therapy with agents known to carry hepatotoxic potential.

## Introduction

Piperacillin/tazobactam (TZP) is a broad-spectrum β-lactam/β-lactamase inhibitor combination widely prescribed for severe infections, including intra-abdominal, pulmonary, and soft tissue infections. Antibiotics are among the most common culprits of drug-induced liver injury (DILI), and TZP has been identified as an important culprit of DILI in hospitalized patients [1,2]. DILI is the leading cause of acute liver failure, with mortality rates in severe cases reaching up to 50% [3]. The diagnosis is challenging because clinical features are nonspecific, and confounding factors such as polypharmacy and underlying illness are common. The prevalence of TZP-induced DILI in hospitalized patients is 0.097% [4]. Although rare, recognition of TZP-associated hepatotoxicity is particularly important, given its frequent use in hospitalized patients. Liver injury due to TZP is most commonly of the cholestatic or mixed type [5]. However, this case is an example of acute hepatocellular injury caused by TZP. We present a case of TZP-induced DILI in a 73-year-old female, emphasizing the importance of vigilance, early diagnosis, and prompt drug withdrawal.

This abstract was previously presented at the American College of Gastroenterology (ACG) 2024 Annual Meeting, October 25-30, 2024, Philadelphia, PA.

## Case presentation

A 73-year-old female with a significant medical history, including hypertension, pulmonary embolism, and a prior right hemicolectomy, was transported from a nursing facility to the emergency department following abnormal laboratory results. The patient had been discharged 10 days earlier after undergoing drainage of a right lower quadrant abscess, which was secondary to an anastomotic leak. Upon discharge, she was prescribed a two-week course of TZP.

Upon arrival, her initial vitals were as follows: blood pressure (BP) of 107/50 mmHg, respiratory rate (RR) of 18 breaths/minute, temperature of 97.52°F, pulse of 111 bpm, and oxygen saturation (SpO2) of 100% on room air. Physical examination revealed a frail, lethargic appearance. Labs revealed sodium at 156 mmol/L, potassium at 1.8 mmol/L, serum creatinine at 1.26 mg/dL, phosphorus at 1.7 mg/dL, total bilirubin at 0.3 mg/dL, aspartate aminotransferase (AST) at 85 U/L, alanine transaminase (ALT) at 35 U/L, normal alkaline phosphatase (ALP), WBC at 5 x 10^3/μL, hemoglobin at 9 g/dL, and platelets at 129 x 10^3/μL (which were within normal limits during her previous admission) (Table 1).

**Table 1:** Admission lab values. Reference range values from the American Board of Internal Medicine, 2025 [6].

Laboratory test	Patient value on admission	Reference range
Sodium (mmol/L)	156	136-145
Potassium (mmol/L)	1.8	3.5-5
White blood cell count (×10³/µL)	5	4.5-11
Serum creatinine (mg/dL)	1.26	0.5-1.1
Phosphorus (mg/dL)	1.7	3-4.5
Total bilirubin (mg/dL)	0.3	0.3-1
Aspartate aminotransferase (U/L)	85	10-40
Alanine transaminase (U/L)	35	10-40
Hemoglobin (g/dL)	9	12-16
Platelets (/µL)	129,000	150,000-450,000

The patient was admitted for intravenous fluid hydration and electrolyte replacement, and TZP was resumed to complete the prescribed two-week course of therapy.

During her hospital stay, there was a progressive increase in liver enzymes and bilirubin levels, which prompted consultation with gastroenterology. The patient underwent a comprehensive infectious and autoimmune workup, including evaluation for Wilson's disease and hemochromatosis; however, all tests returned negative results. Abdominal imaging, including both ultrasound and contrast-enhanced CT scan of the abdomen and pelvis, revealed evidence of hepatic steatosis.

**Figure 1:**
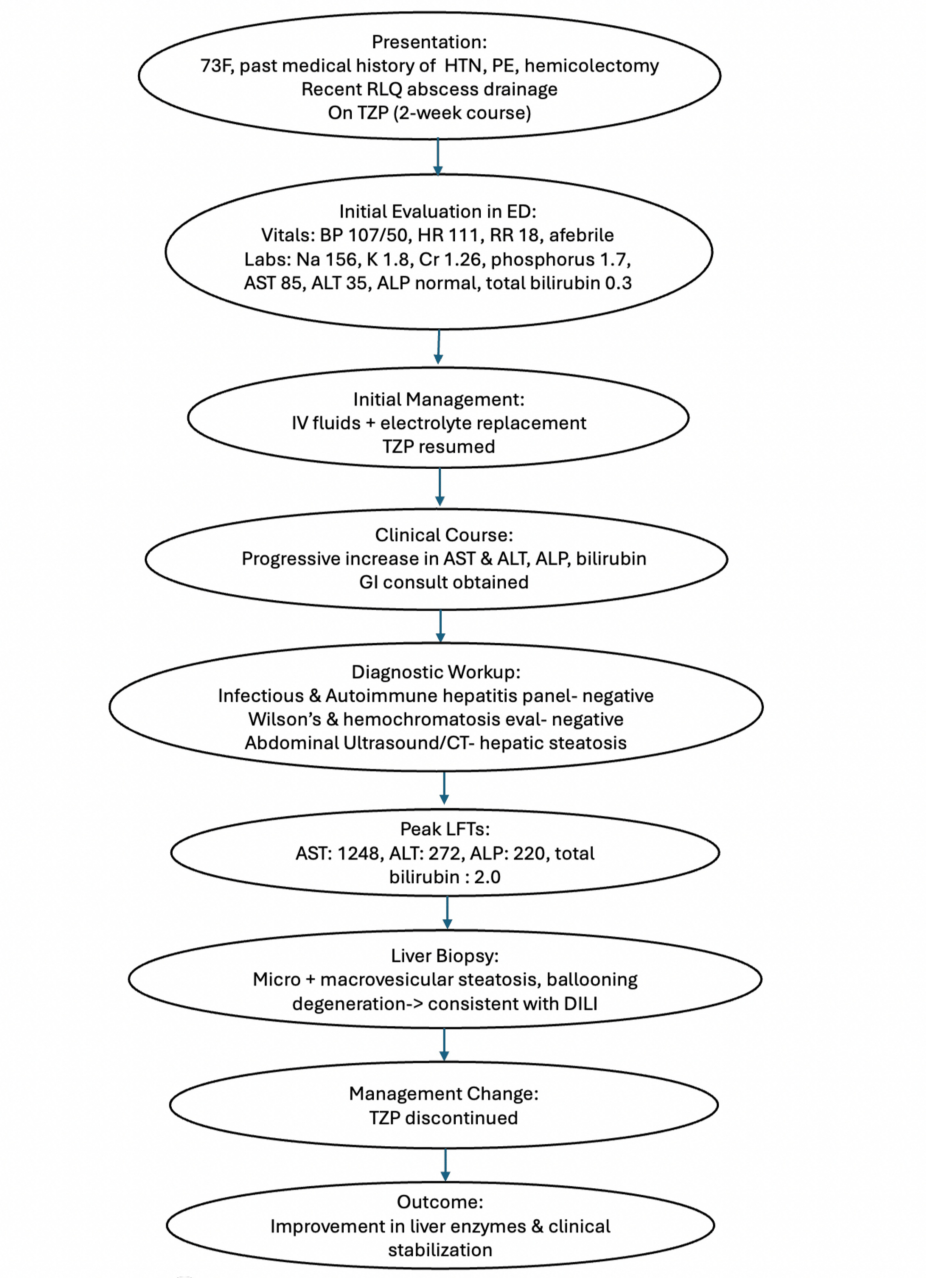
Clinical course flowchart. HTN: hypertension; 73F: 73-year-old female; PE: pulmonary embolism; RLQ: right lower quadrant; TZP: piperacillin/tazobactam; AST: aspartate aminotransferase; ALT: alanine transaminase; ALP: alkaline phosphatase; DILI: drug-induced liver injury; BP: blood pressure; HR: heart rate; RR: respiratory rate; GI: gastrointestinal; LFT: liver function tests.

Due to significant elevation in liver enzymes (AST = 1248 U/L, ALT = 272 U/L, ALP = 220 U/L) and total bilirubin (2 mg/dL) (Table 2), a liver biopsy was performed, demonstrating microvesicular and macrovesicular steatosis, as well as ballooning degeneration, indicative of DILI. These biopsy results also assisted in ruling out hepatic hypoperfusion as a possible cause of the patient's elevated liver enzymes since the biopsy lacked centrilobular necrosis, the characteristic finding in hypoperfusion injuries of the liver. Subsequently, TZP was discontinued, resulting in an improvement in liver enzyme levels. Two tools used to calculate the likelihood of a liver injury being due to a specific drug are the Roussel Uclaf Causality Assessment Method (RUCAM) score and the Naranjo scale [5]. This patient's RUCAM score was 7, and the Naranjo scale score was 5. Both scores indicate that the causality assessment of TZP as the cause of this patient's liver injury was probable. Figure 1 summarizes the clinical course of this patient.

**Table 2:** Liver function tests. Reference range values from the American Board of Internal Medicine, 2025 [6].

Liver function test	Patient peak value	Reference range
Aspartate aminotransferase (U/L)	1248	10-40
Alanine transaminase (U/L)	272	10-40
Alkaline phosphatase (U/L)	220	30-120
Total bilirubin (mg/dL)	2	0.3-1

## Discussion

DILI caused by TZP is rare, but as this case demonstrates, it can cause acute, significant liver injury. Once recognized, DILI first requires cessation of the offending agent with hospitalization and liver transplant to follow, if necessary, based on the degree of injury. The outcome greatly depends on how quickly the drug responsible is recognized and withdrawn. Therefore, although DILI is a diagnosis of exclusion, clinicians should have a high degree of suspicion of DILI in cases of acute liver injury. In fact, DILI is the most common cause of acute liver failure in the United States [7].

The liver has the responsibility of modifying and detoxifying substances and thus is put in a position to be harmed directly by drugs and their metabolites or by immune processes triggered by the same drugs. DILI can be caused by either of these processes or by a mixture of both direct toxicity and immune-mediated injury. It is suggested that the cause of liver injury induced by piperacillin is due to hypersensitivity or allergy, while the cause of liver injury due to tazobactam is more uncertain [5].

Altered liver enzymes are non-specific but are a hallmark for diagnosing DILI [8]. Physical exam findings present in DILI can include jaundice, encephalopathy, abdominal pain, and, as seen in this case, fatigue. A careful drug history should be obtained, focusing especially on new drugs introduced in the last three months. If DILI is suspected based on elevated liver enzymes, physical findings, and drug history, the RUCAM can be used to determine the likelihood that the hepatic injury is due to a specific medication [5]. This includes calculating the R ratio, which determines if the pattern of injury is hepatocellular, cholestatic, or mixed. In this case, based on the patient's elevated ALT and ALP levels, the injury pattern was determined to be hepatocellular. Hepatocellular cases of TZP-induced DILI have been noted, but are less common than cholestatic or mixed types [5]. The RUCAM score is a tool that takes into account several factors, including the R ratio, the time of onset, the course of the illness, risk factors such as a history of alcohol use and age over 55, concomitant medications, evaluation for non-drug causes, prior knowledge of the hepatotoxicity of the suspected drug, and the response to readministration. It categorizes the likelihood that the liver injury was caused by a specific drug as "excluded," "unlikely," "probable," or "highly probable" [5]. In this case, the patient's RUCAM score of 7 falls within the "probable" range. The patient's thrombocytopenia is also notable because, although platelet count is not a major diagnostic marker in DILI, a decrease in platelets can indicate more severe liver injury, poor prognosis, and even increased probability of impending liver or multi-organ failure [9].

In this case, the patient had been taking TZP for 10 days, had become acutely frail and lethargic, and then had increasingly elevated liver enzymes throughout her hospital stay. She was over the age of 55, had recently started one new medication, and autoimmune and infectious causes of liver injury were ruled out. It should be noted that although response to re-administration is included in the RUCAM score, it is rarely done and is only useful in cases where a drug is inadvertently given again, and liver injury is noted a second time. TZP hepatotoxicity is thought to have a drug-sensitivity or hypersensitivity mechanism of injury and can develop days to weeks after initiation of treatment [5].

Imaging studies and possibly liver biopsy are other important steps in investigating DILI. In this case, the elevated liver enzymes prompted imaging and a biopsy that revealed steatosis and ballooning degeneration suggestive of DILI. Steatosis and DILI have a bidirectional relationship that can complicate definitive diagnosis. While steatosis can be due to direct injury from a drug, it can also be due to chronic pre-existing changes [10]. Additionally, pre-existing steatosis can predispose patients to DILI [10]. In this case, the RUCAM score combined with the improvement of the liver injury following cessation of a specific medication allowed for the diagnosis of DILI. The cessation of TZP resulted in improvement of liver enzymes, indicating that TZP was the offending agent. As seen in this case, DILI due to TZP most commonly has a good outcome in which cessation of the drug leads to resolution and return to baseline liver function tests within a few weeks [5]. Still, there is a wide range of severity, and cases of chronic cholestatic injury due to TZP have been reported [5].

## Conclusions

This case highlights the critical importance of monitoring liver function in patients receiving TZP, particularly in those with complex medical histories and extended courses of antibiotic therapy. Early recognition of DILI and the prompt discontinuation of the offending agent are essential for minimizing adverse effects and facilitating liver recovery. As seen here, liver biopsy is often a helpful step to reaching answers in cases of DILI. This case also emphasizes the utility of assessment tools such as the RUCAM score to assist in confident diagnosis or exclusion of DILI. The liver biopsy and RUCAM score, coupled with the improvement of the patient's liver function tests following cessation of TZP, are what allowed for the diagnosis of DILI in this case. This case also serves as a reminder for clinicians to maintain a high index of suspicion for DILI when evaluating unexplained liver enzyme elevations during antibiotic therapy, and to consider alternative agents when risk factors for hepatotoxicity are present.
